# Bioengineered silkworm model for expressing human neurotrophin-4 with potential biomedical application

**DOI:** 10.3389/fphys.2022.1104929

**Published:** 2023-01-04

**Authors:** Wenchang Zhang, Zhiqing Li, Weiqun Lan, Hao Guo, Feng Chen, Feng Wang, Guanwang Shen, Qingyou Xia, Ping Zhao

**Affiliations:** ^1^ State Key Laboratory of Silkworm Genome Biology, Biological Science Research Center, Southwest University, Chongqing, China; ^2^ Integrative Science Center of Germplasm Creation in Western China (CHONGQING) Science City & Southwest University, Chongqing, China; ^3^ Chongqing Key Laboratory of Sericultural Science, Chongqing Engineering and Technology Research Center for Novel Silk Materials, Southwest University, Chongqing, China

**Keywords:** bombyx mori, human neurotrophic-4, silk material, biomedical application, silk gland bioreactor

## Abstract

Neurotrophin-4 (NT-4) is a neurotrophic factor that plays important roles in maintaining nerve cell survival, regulating neuronal differentiation and apoptosis, and promoting nerve injury repair. However, the source of sufficient NT-4 protein and efficient delivery of NT-4 remain a challenge. This study aims to express an activated human NT-4 protein in a large scale by genetically engineering silk gland bioreactor of silkworm as a host. We showed that the expression of human NT-4-functionalized silk material could promote proliferation of mouse HT22 cells when compared to the natural silk protein, and no obvious cytotoxicity was observed under the conditions of different silk materials. Importantly, this functional silk material was able to induce the potential differentiation of HT22 cells, promote peripheral neural cell migration and neurite outgrowth of chicken embryo dorsal root ganglion (DRG). All these results demonstrated a high bioactivity of human NT-4 protein produced in silk gland. Therefore, based on the silkworm model, the further fabrication of different silk materials-carrying active NT-4 protein with good mechanical properties and great biocompatibility will give promising applications in tissue engineering and neurons regeneration.

## Introduction

Neurotrophins are a family of growth factors that can regulate neuron development, differentiation, and survival in the central and peripheral nervous systems (Bibel and Barde, 2000; Huang and Reichardt, 2001). There are four main neurotrophins including Nerve growth factor (NGF), Brain-derived neurotrophic factor (BDNF), Neurotrophin-3 (NT-3) and NT-4. Among them, NT-4 is a recently identified novel neurotrophin and its function remains largely uncovered. NT-4 was first discovered in *Xenopus laevis* and viper and has been subsequently found in humans and other mammals (Hallböök et al., 1991; Ip et al., 1992; Hafidi, 1999; Shen et al., 2010; Guo et al., 2017). Human NT-4 protein is 210 amino acids in length and consists of three regions including signal peptide, precursor peptide and mature body. The mature body is the active form of NT-4 protein, which is composed of 129 amino acid residues and has a molecular weight of 14 kDa (Ip et al., 1992). Recent studies have reported that NT-4 possesses a wide range of functions, mainly through binding with tropomyosin receptor kinase receptor (TrkB) and p75 neurotrophin receptor (p75NTR) to regulate downstream signaling pathways and then participate in maintaining the survival of nerve cells, regulating the differentiation and apoptosis of neurons, and promoting the repair of nerve injury (Bothwell, 1991; Conover et al., 1995; Liu et al., 1995; Agerman et al., 2000; Ernfors, 2001; Runge et al., 2012).

In mammals, NT-4 treatment of neural stem cells isolated from mouse embryos can reduce the expression of neural marker genes, by contrast, it increases the number of neural progenitor cells, which indicates that neural stem cells begin to differentiate into neuron cells with the participation of NT-4 protein (Shen et al., 2010). The application of NT-4 in injured rat sciatic nerve can promote the axonal regeneration and increase the thickness of myelin and diameter of axonal (Yin et al., 2001), demonstrating a great potential of NT-4 protein in repairing nerve injury. In addition to playing an important role in the nervous system, NT-4 is reported to be involved in the regulation of ovarian follicle formation. For instance, the addition of NT-4 to cultured human fetal ovaries enhances follicular assembly (Farhi et al., 2011). Whereas deletion of NT-4 in mice results in damaged follicular tissue, increased oocyte death, and severe ovarian abnormalities (Ojeda et al., 2000). Due to the great demand of NT-4 in biomedical application, the natural source of NT-4 protein cannot meet the needs of basic research and medical application (Machalinska et al., 2013). Therefore, mass production of recombinant NT-4 protein instead of natural protein to exert its bioactivity has arisen great concerns of researchers. To data, the commercial active NT-4 protein is mainly obtained and purified from expression systems like *Escherichia coli* and *Spodoptera frugiperda* sf21 cells, however, the expression of NT-4 in other systems remains to be explored.

The silkworm *Bombyx mori* is one of the important economic insects that has been artificially bred and raised, and its economic value mainly comes from its silk gland, the organ that can efficiently synthesize abundant silk proteins and secrete them into silk cocoon (International Silkworm Genome, 2008; Xia et al., 2014). With the efforts on developing silkworm transgenic technology, it has formed a complete set of technical processes from vector construction to embryo microinjection and screening of transgenic positive individuals (Tamura et al., 2000; Tomita, 2011). Based on the silk gland-specific expression genes, sericin and fibroin promoters have been established to express exogenous proteins specifically localized in middle silk gland (MSG) and posterior silk gland (PSG), respectively, which has established the silkworm as an important host to express foreign proteins (Kurihara et al., 2007; Tomita et al., 2007). Until now, most foreign proteins with various biological functions and potential biomedical applications have been successfully expressed in silk gland of silkworm, such as human lactoferrin, human platelet derived growth factor, human type III procollagen, human serum albumin, human acidic fibroblast growth factor, and others (Tomita et al., 2003; Ogawa et al., 2007; Wang et al., 2015; Chen et al., 2018; Xu et al., 2021). Therefore, make the best of silk gland *via* transgenic silkworm has also been an ideal bioreactor for producing recombinant drug proteins in a large scale to meet the growing clinical needs and applications.

In the present work, by using the silk sericin expression system, we constructed a transgenic silkworm strain expressing the foreign human NT-4 protein in MSG. It showed that NT-4 protein was highly expressed in silkworm MSG. After calculation, the NT-4 content in per Gram of silkworm cocoon was about 0.4 mg, supporting this efficient expression system of silkworm silk gland. The use of silkworm cocoon extract containing NT-4 to culture mouse HT22 cells was able to promote cell proliferation and upregulate the expression of neuronal and astrocyte marker genes, as well as the expression of genes related to myelination in HT22 cells. Moreover, chicken embryo dorsal root ganglion (DRG) was isolated and treated by NT-4 protein, and it was found that the silk material containing NT-4 protein could effectively promote the outward migration of DRG cells. All these results further demonstrated the potential of silk gland bioreactor as a useful strategy for efficient and large-scale production of active NT-4 protein, which could be fabricated to generate various biomaterials by using NT-4 expressing silk cocoon for biomedical application.

## Materials and methods

### Silkworm strain and cell line

The silkworm D9L strain fed by fresh mulberry leaves or artificial diet was applied to generate transgenic expression of recombinant human NT-4 protein (Accession No. NP_001382418). The mouse Hippocampal neuronal cell HT22 cell line (Procell, Wuhan, China) was cultured with Dulbecco’s Modified Eagle’s Medium (DMEM, Gibco, Waltham, MA, United States) containing 10% (v/v) fetal bovine serum (FBS, Gibco, Waltham, MA, United States) at 37°C in a 5% CO_2_ atmosphere.

### Plasmid construction

The optimized human NT-4 gene according to the silkworm codon bias (Supplementary Table S1) was synthesized by BGI company (Shenzhen, China). Primers used for gene amplification are listed in Supplementary Table S2. The NT-4 gene was digested by BamHI/NotI and cloned into Sericin-1 expression vector pSL1180 [Hr3Ser1PSer1PA] (Wang et al., 2013). The plasmid containing NT-4 gene was further digested by AscI and inserted into transgenic backbone vector piggyBac [3xP3DsRed] to obtain the transgenic expression plasmid piggyBac [DsRed,NT-4]. The plasmid was verified by sequencing (BGI, Shenzhen, China).

### Generation of transgenic silkworm

Generation of transgenic silkworm was performed according to a previously reported method (Tamura et al., 2000). Briefly, the plasmids for microinjection were extracted by using the QIAGEN Mini kit (Qiagen, Hilden, Germany). After mixture of the target piggyBac plasmid and the pHSP70PIG helper plasmid at a mole ratio 1:1 with a final concentration of 500 ng/μL, the mixture plasmids were microinjected into the non-diapause silkworm embryos within 2 h after oviposition by a microinjector (Eppendorf, Hamburg, Germany). The hatched G0 larvae were fed to oviposit the G1 eggs, and the positive G1 eggs were fluorescently screened through eyes where DsRed was specifically expressed by using an Olympus SZX12 fluorescence stereomicroscope (Olympus, Tokyo, Japan). DsRed fluorescence was specifically stimulated by the transgenic silkworm line expressing NT-4 protein in MSG. The G1 positive individuals were named as NT-4, reared to the moth stage, and crossed or backcrossed with moths of wild type silkworm to generate stable transgenic silkworm. The cocoon from transgenic silkworm expressing NT-4 in MSG were collected for NT-4 expression analysis.

### Crude extraction of NT-4 from the cocoon silk

The cocoon silk from the NT-4 transgenic silkworm were ground and extracted with a concentration of 30 mg/ml by using the lysis buffer (50 mM Tris-HCl, 8 M urea, pH 7.0) at 4°C (Wang et al., 2015). The crude extract was then centrifuged at 20,000 g for 20 min, and the supernatant was filtrated with 0.45 μm Durapore®PVDF Membrane (Millex®-HV). Further the extract was ultrafiltered with a 100 K ultrafiltration tube (Millipore, Temecula, CA, United States), the filtered liquid was collected. To concentrate the collected liquid, a 3 K ultrafiltration tube (Millipore, Temecula, CA, United States) was used, and the liquid in the retention tube was collected. The concentrated solution in the retention tube was dialyzed in cellulose dialysis membrane (MWCO 14,000 Da, Sangon, China) against the dialysis solution (50 mM Tris-HCl, pH 7.0, 250 mM NaCl) for 3 days with the replacement of dialysis solution every 12 h to remove the urea. The dialysis-completed extract containing the recombinant NT-4 protein was dispensed into small volume according to the needs of the experiment, and stored at −80°C.

### Quantitative real-time PCR analysis

To detect the expression of the exogenous NT-4, the total RNAs from middle silk gland of WT and transgenic silkworm on day 6 of the 5th instar larvae were extracted using a Total RNA Kit (Omega, Biel/Bienne, Switzerland). 2 μg of each sample was used for reverse transcription to obtain the corresponding cDNA by a GoScript Reverse Transcription System (Promega, Madison, WI, United States). Subsequently, an equal amount of the cDNA was subjected for qRT-PCR assay using the SYBR Premix Ex TaqTM II kit (TaKaRa & Clontech, Dalian, China) on the Applied Biosystems 7,500 Fast Real-Time PCR System (Applied Biosystems, Foster City, CA, United States) with a program consisting of an initial denaturing step of 30 s at 95°C and 40 amplification cycles consisting of 5 s at 95°C followed by 30 s at 60°C. The transcriptional expression levels of the exogenous NT-4 in the WT silkworm and the NT-4 transgenic silkworm were normalized to the corresponding internal control of *eukaryotic translation initiation factor 4A* (*eIF-4a*) (Wang et al., 2015). All experiments were independently performed with three biological replicates, and the relative mRNA expression levels were calculated using the 2^−△△CT^ method. All primers used for qRT-PCR are listed in Supplementary Table S2.

To analyze the expression of differentiation genes in HT22 cells, total RNA from HT22 cells treated with different cocoon extracts were isolated using a Total RNA Kit, and subjected for qRT-PCR assay. Primers of *Glial fibrillary acidic protein* (*GFAP*), *Neuron specific enolase* (*NSE*), *Neuronal class Ⅲ beta-Tubulin* (*TUBB3*), *Neuron microtubule-associated protein 2* (*MAP2*), *Myelin protein zero* (*MPZ*), *Myelin basic protein* (*MBP*), and *Glyceraldehyde-3-phosphate dehydrogenase* (*GAPDH*) are listed in Supplementary Table S2.

### Western blot analysis

Cocoon from NT-4 and WT were frozen in liquid nitrogen for 1 minute, powdered, and then the powder with a concentration of 30 mg/ml was suspended by lysis buffer containing 50 mM Tris-HCl and 8 M urea at pH 7.0 under 80°C for 30 min. The extracts were centrifuged at 20,000 g for 5 min to collect the supernatant samples. After quantification of proteins in cocoon extraction by Bradford method, the expression level of NT-4 protein was detected by western blot analysis. 30 μL of protein samples were separated on the sodium dodecyl sulfate-polyacrylamide gel electrophoresis (SDS-PAGE) and electronically transferred to the polyvinylidene fluoride (PVDF) membrane, followed by the immunoreaction with anti-NT-4 antibody (Abcam, Cambridge, United Kingdom) at a dilution of 1:10,000 and secondary horse radish peroxidase (HRP)-labeled goat anti-rabbit IgG (Beyotime, Shanghai, China) at a dilution of 1:20,000. The immunoreacted PVDF membrane was finally visualized using chemiluminescence ECL western blotting kit (Amersham Biosciences, Little Chalfont, United Kingdom), and the images were recorded using a Chemiscope Series (Clinx Science Instruments, Shanghai, China).

For MBP detection, HT22 cells treated with different cocoon extracts were collected by trypsinization, and RIPA cell lysate (Beyotime, Shanghai, China) was added to the cells for protein extraction. 30 μL of protein samples were separated on the SDS-PAGE and electronically transferred to the PVDF, followed by the immunoreaction with anti-MBP antibody (Beyotime, Shanghai, China) and anti-GAPDH antibody at a dilution of 1:10,000, and secondary HRP-labeled goat anti-rabbit IgG at a dilution of 1:20,000. The images were acquired using a Chemiscope Series.

### Quantification analysis of NT-4

To quantify the expression of NT-4 protein, NT-4 in MSG with the highest expression level was selected and separated by the SDS-PAGE with a gel concentration of 12%. For the Coomassie brilliant blue (CBB) analysis, 30 μL of the protein samples, together with 125 ng, 250 ng, and 500 ng of BSA standard (Beyotime, Shanghai, China) were used. For the Western blot analysis, 30 μL of the protein samples, together with 200 ng, 300 ng, and 400 ng of NT-4 standard (Abcam, Cambridge, United Kingdom) were used. After electrophoresis, the gels were stained with CBB R250 or transferred to the Western blot analysis. The content of NT-4 in the cocoon shell weight was quantified by analyzing the intensity of the extracted NT-4 with BSA standard on the CBB and NT-4 standard on the Western blot using ImageJ software (https://imagej.nih.gov/ij/).

### Cell proliferation assay

The HT22 cells were seeded in 96-well or 24-well plates and incubated with the extract of NT-4 silkworm cocoon or WT silkworm cocoon at a dosage of 100 ng/ml for 24 h. Cell proliferation of HT-22 was measured using the Cell Counting Kit-8 (Beyotime, Shanghai, China) and the BeyoClick™ EdU-488 (Beyotime, Shanghai, China) according to the manufacturers’ protocol. For the CCK-8 assays, HT22 cells treated by different extracts were incubated with CCK-8 solution (10 μL per well of a 96-well plate) at 37°C for 1 h, and the absorbance was measured at 450 nm in a Glomax Multi Detection System (Promega, Madison, WI, United States). For the EdU staining, HT22 cells after treatment were labeled by EdU at a concentration of 2 μM at 37°C for 2 h. The labeled cells were immediately fixed with 4% paraformaldehyde (Beyotime, Shanghai, China) in PBS and permeabilized by 0.5% Triton^®^ X-100 (Beyotime, Shanghai, China), followed by EdU detection using Alexa Fluor 488 and the proliferated cells were photographed under a fluorescence microscope (Olympus, Tokyo, Japan).

### Immunofluorescence staining

The HT22 cells were seeded in 24-well plate and incubated with the extract of NT-4 silkworm cocoon or WT silkworm cocoon at a dosage of 100 ng/ml for 72 h. The 4% paraformaldehyde was added to fix the cells. After the fixation, the cells were permeabilized with 0.5% Triton^®^ X-100 at room temperature for 20 min, and washed 3 times with PBS. The fixed cells were blocked in 5% goat serum (Beyotime, Shanghai, China) at room temperature for 30 min, and incubated with primary antibody against GFAP (Beyotime, Shanghai, China) at a dilution of 1:10,000 overnight at 4°C. After extensive wash, the cells were incubated with a Cy3-labeled secondary antibody at 1:20,000 (Beyotime, Shanghai, China) for 1 h, and the nuclei were stained by DAPI (Beyotime, Shanghai, China) for 10 min. Images were acquired using a fluorescence microscope (Olympus, Tokyo, Japan).

### Dissection of chick embryonic dorsal root ganglion

The chick embryonic dorsal root ganglion (DRG) was isolated as described previously (Wood, 1976; McDonald et al., 2005). Briefly, the chicken embryos (Chongqing, China) were cultured in a 37°C incubator until the 10th day. The shell of chicken embryos and dissection tools were fully sterilized with 75% ethanol. The chicken embryos were pulled out using dissecting forceps and placed in a petri dish. Scissors were used to decapitate the embryo and discard the head. With the embryo on its back, the spinal cord of all the visceral tissues and organs were dissected using the forceps and cleared by dissection media. Under a dissecting microscope (Olympus, Japan), the DRG from the spinal cord was harvested and cultured in RPMI-1640 complete medium (Gibco, United States). The above sampling operations were all performed in a sterile environment.

### Identification of the DRG

The cells that migrated out after 48 h of DRG culture were taken, and 4% paraformaldehyde (Beyotime, Shanghai, China) was added to fix the cells. After the fixation, the cells were washed 3 times with PBS and permeabilized with 0.5% Triton^®^ X-100 (Beyotime, Shanghai, China) at room temperature for 20 min. After aspirating the cleaning solution, 5% goat serum (Beyotime, Shanghai, China) was added and blocked at room temperature for 30 min. Then, anti-GFAP (Beyotime, Shanghai, China) as the primary antibody at a dilution of 1:10,000 was incubated overnight at 4°C and Cy3-labeled secondary antibody (Beyotime, Shanghai, China) at a dilution of 1:20,000 was incubated at room temperature without light for 1 h. After excessively washing with PBS, the cell nuclei were stained by DAPI and observed under a fluorescence microscope (Olympus, Tokyo, Japan).

### Effect of NT-4 on the DRG

The freshly obtained DRGs were placed in a 24-well cell culture plate with one DRG per well. Each well was added with 200 μL of culture medium and an equal volume of PBS, WT cocoon extract, or NT-4 cocoon extract. The DRG cell migration and neurite outgrowth was observed under a microscope and photographed at different times. Three replicates were performed for each treatment.

### Statistical analysis

Data are presented as the mean ± standard deviation (SD) of three independent biological replicates. Statistical significance (*p*-value) was analyzed by the Student’s *t*-test. Statistical significance is denoted as follows: *p < 0.05, **p < 0.01, and ***p < 0.001.

## Results

### Generation of transgenic silkworm for human NT-4 expression

**FIGURE 1:**
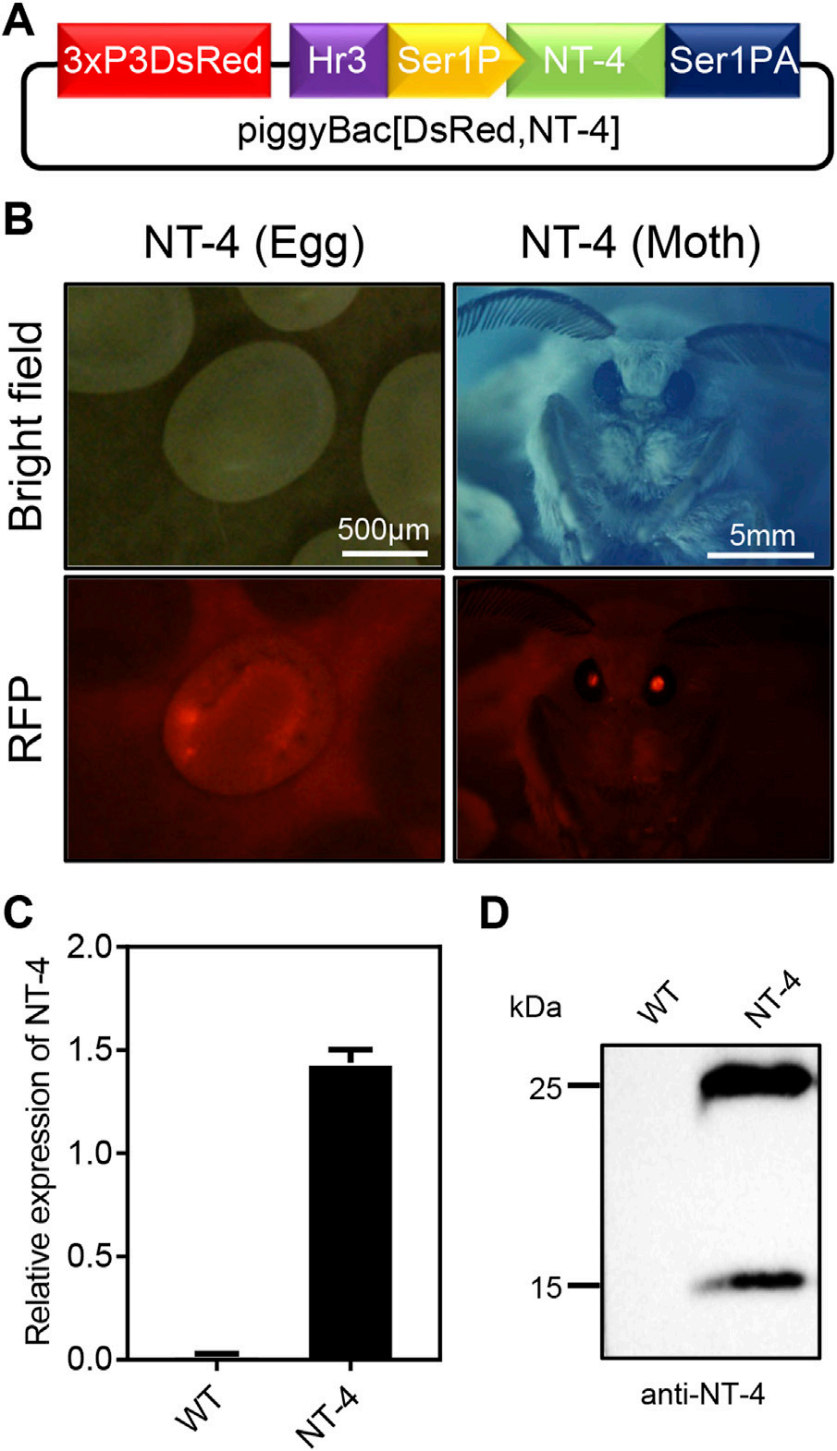
Generation of transgenic silkworm for human NT-4 expression. **(A)** A schematic diagram of piggyBac [DsRed,NT-4] transgenic expression vector in MSG of silkworm. **(B)** Screening for the positive G1 eggs and moth exposed under RFP fluorescence (red), scale bar is 500 µm and 5 mm, respectively. **(C)** The mRNA expression of NT-4 by qRT-PCR analysis. **(D)** Protein analysis of NT-4 by Western blotting. The upper and lower bands were predicated as full size and mature peptides of NT-4, respectively.

In order to express the human NT-4 protein in silkworm, we first constructed the piggyBac transgenic expression vector of human NT-4 by using the middle silk gland expression system according to the previous report (Figure 1A) (Wang et al., 2013). The expression plasmid of piggyBac [DsRed,NT-4] was mixed with the transposase expression plasmid pHA3PIG according to the mass ratio of 1:1, and then injected into the early embryos of the non-diapause silkworm. The hatched larvae (G0 generation) were fed with mulberry leaves or artificial diet, and the adults were mated and laid eggs. The obtained G1 offspring eggs were developed at 25°C until the 7th day. The positive transgenic individuals with specific excitation of red fluorescence in the ocelli of the body pigmentation stage of the embryos and the compound eyes of the moths (Figure 1B) were screened by a fluorescence microscope, suggesting the successful transformation of hereditable expression of NT-4 in silkworm.

To further confirm the expression of NT-4 in the silk gland of silkworm, total RNA from the MSG of silkworm were extracted and the expression of NT-4 were detected by qRT-PCR. As shown in Figure 1C, NT-4 had been successfully transcribed in the transgenic silkworm. Meanwhile, proteins from the transgenic NT-4 and WT cocoon were extracted respectively, and their expression was detected by western blotting after protein quantification using Bradford method. The results showed that NT-4 protein was highly expressed and secreted into silkworm cocoon in the expression system of middle silk gland (Figure 1D). It was interestingly found that there were two specific bands near 25 kDa and 15 kDa. Molecular weight of 25 kDa was the full size of NT-4 protein, and we speculated that the 15 kDa might be the active form of NT-4 (Ip et al., 1992). All these data demonstrated that we have established the transgenic silkworm for expressing NT-4.

### Quantitative analysis of NT-4 expression in transgenic silkworm

**FIGURE 2:**
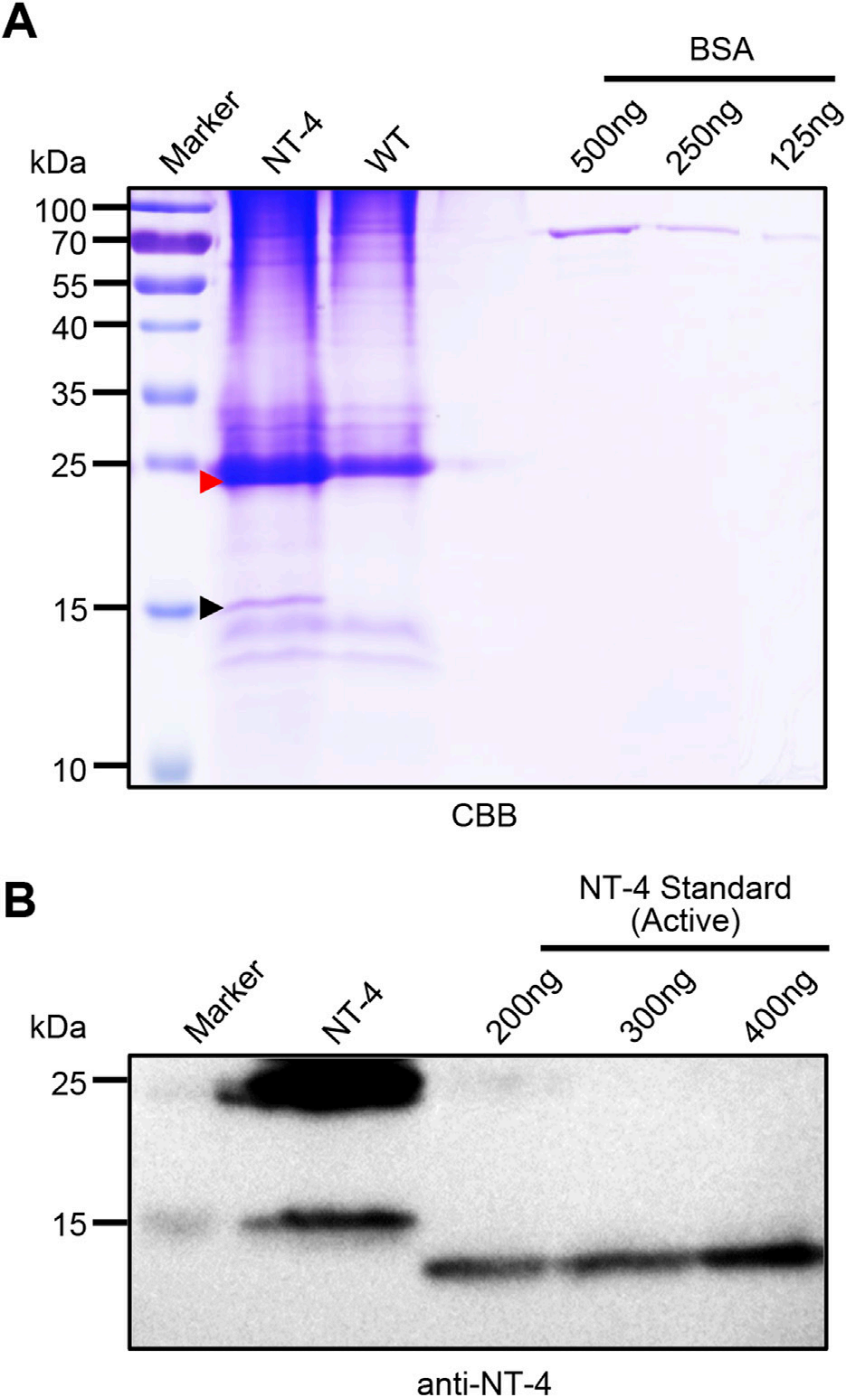
Quantitative analysis of NT-4 expression in transgenic silkworm. **(A)** SDS-PAGE analysis of the crude extracts from NT-4 and WT silkworm cocoon. BSA standard with different contents of 125 ng, 250 ng, and 500 ng were used to quantitate the recombinant NT-4 concentration. The red and black arrowheads point to the full size and mature peptides of NT-4, respectively. **(B)** Western blotting analysis of the crude extracts from NT-4 silkworm cocoon. Active NT-4 standard with different contents of 200 ng, 300 ng, and 400 ng were used to quantitate the recombinant NT-4 concentration.

To analyze the content of NT-4 protein in transgenic silkworm cocoon, we extracted the cocoon proteins from the transgenic silkworm and analyzed by SDS-PAGE and western blotting. Gel staining by CBB showed that there were significantly visible protein bands near 25 kDa and 15 kDa. Although the 25 kDa band was mixed with the silkworm endogenous proteins, it was still obvious that the protein contents in NT-4 extract was much higher than WT. In particular, the specific protein at 15 kDa in NT-4 was distinct from WT (Figure 2A). On the other word, this was consistent with the Western blotting result in Figure 1D. We used the gray values of 125 ng, 250 ng, and 500 ng of BSA protein bands to draw the standard curve and evaluate the content of NT-4 protein. The content of NT-4 protein in the cocoon from the transgenic silkworm strain was about 0.4 mg/g of the cocoon shell weight (Figure 2A). We also purchased a commercial standard of mature NT-4 protein and estimated the protein contents by western blotting analysis. It was shown that the mature NT-4 standard was similar to the 15 kDa form activated in silkworm, while the slight increase of activated NT-4 might be due to the potential modification. All these results confirmed the presence of two specific bands of full and mature NT-4 peptides in silkworm expression system. We used different concentrations of 200 ng, 300 ng, and 400 ng of NT-4 standard proteins to draw the standard curve by the gray value. The result showed that per Gram of NT-4 strain silkworm cocoon could produce approximately 0.4 mg NT-4 protein (Figure 2B), which was consistent with the quantitation of CBB staining (Figure 2A).

### NT-4 promoted the proliferation of HT22 cells

**FIGURE 3:**
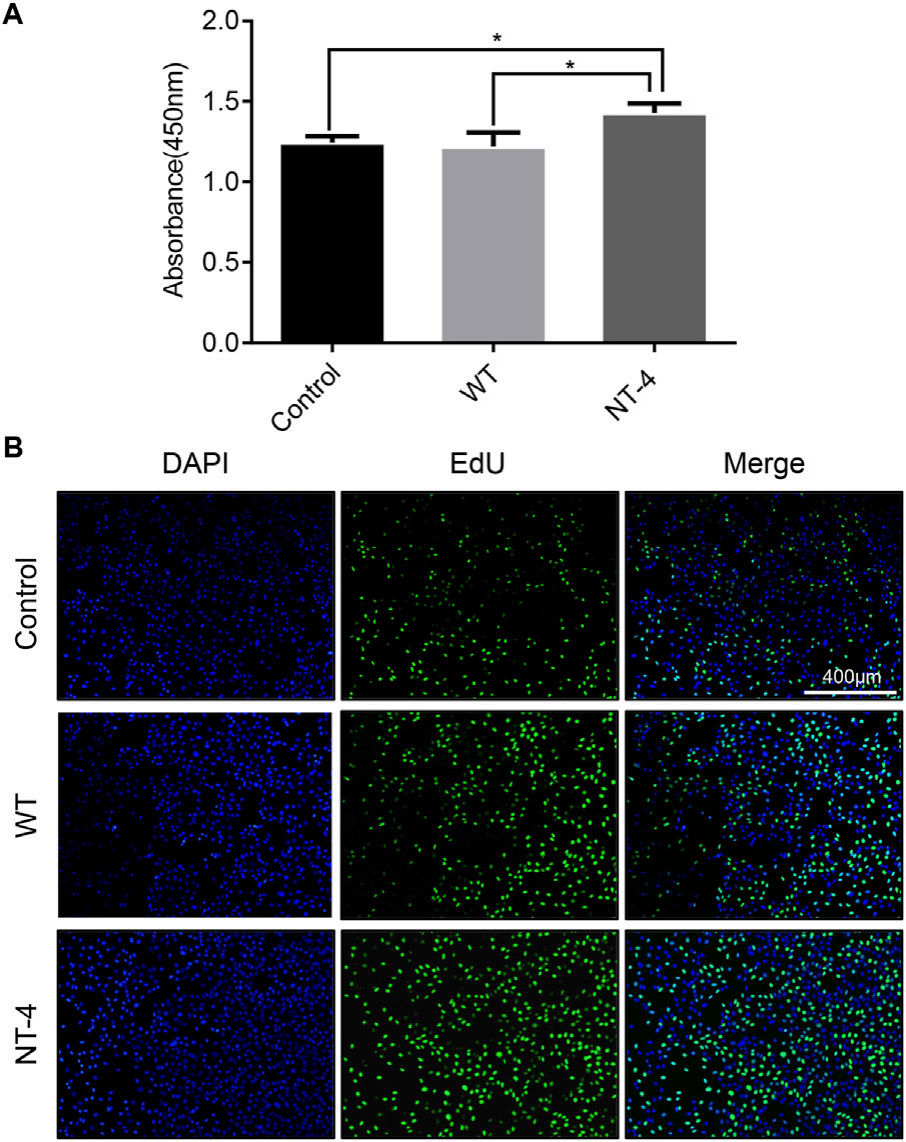
NT-4 promoted the proliferation of HT22 cells. **(A)** CCK-8 assay of WT and NT-4 extracts on HT22 cell proliferation after 24 h treatment. For the significant analysis: *p < 0.05. **(B)** EdU-488 staining was used to analyze HT22 cells undergoing DNA replication and proliferation by different treatments (green). The nuclei DNA was counterstained with DAPI (blue). Scale bar is 400 μm.

To identify whether the silk material containing NT-4 protein extracted from transgenic cocoon possessed the bioactivity, we first analyzed its effects on cell proliferation in HT22 cells, a cell line of mouse hippocampal neurons (Zhang et al., 2018). The starved HT22 cells were treated with silkworm cocoon extract containing NT-4 protein and WT cocoon extract. The CCK-8 proliferation assay was performed in HT22 cells after culturing of NT-4 and WT for 24 h. The results showed that the OD absorbance in treatment group containing NT-4 protein was significantly higher than that of the WT and control groups (Figure 3A), indicating that NT-4 protein expressed in silkworm had the bioactivity of promoting cell proliferation. Additionally, EdU incorporation was also used to monitor the newly synthesized DNA by cells within the same treatments. It was shown that only a small number of HT22 cells from the control group exhibited green fluorescence signals, by contrast, more cells treated by NT-4 emitted intensive signals (Figure 3B), which was consistent with that of CCK-8 result. These data thus revealed that the NT-4 protein expressed in silk gland possessed the ability of promoting cell proliferation and DNA synthesis of neuron cells.

### NT-4 promoted the expression of neuronal marker genes in HT22 cells

**FIGURE 4:**
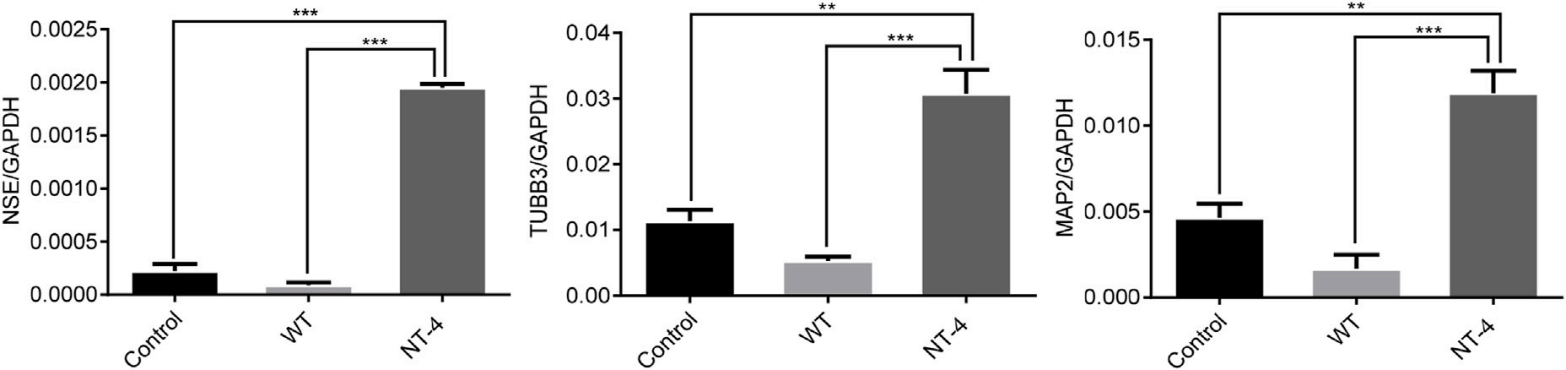
NT-4 promoted the expression of neuronal marker genes in HT22 cells. The mRNA expression of neuronal marker genes including *NSE*, *TUBB3*, and *MAP2* in HT22 cells were analyzed by qRT-PCR after the treatments with WT and NT-4 extracts. *GADPH* was used as the internal control. For the significant analysis: **p < 0.01 and ***p < 0.001.

NT-4 has potential trophic effects on subpopulations of neurons (Yin et al., 2001). We then evaluated the expression of the neuronal-specific marker genes in HT22 cells upon the NT-4 treatment. The qRT-PCR results showed that the cocoon extract containing NT-4 protein could significantly upregulate the expression of neuronal marker genes including *NSE* (Lee et al., 2010), *TUBB3* (Alexander et al., 1991), and *MAP2* (Izant and McIntosh, 1980) when compared to the control and WT silkworm cocoon extract (Figure 4).

### NT-4 contributed to myelination process in HT22 cells

**FIGURE 5:**
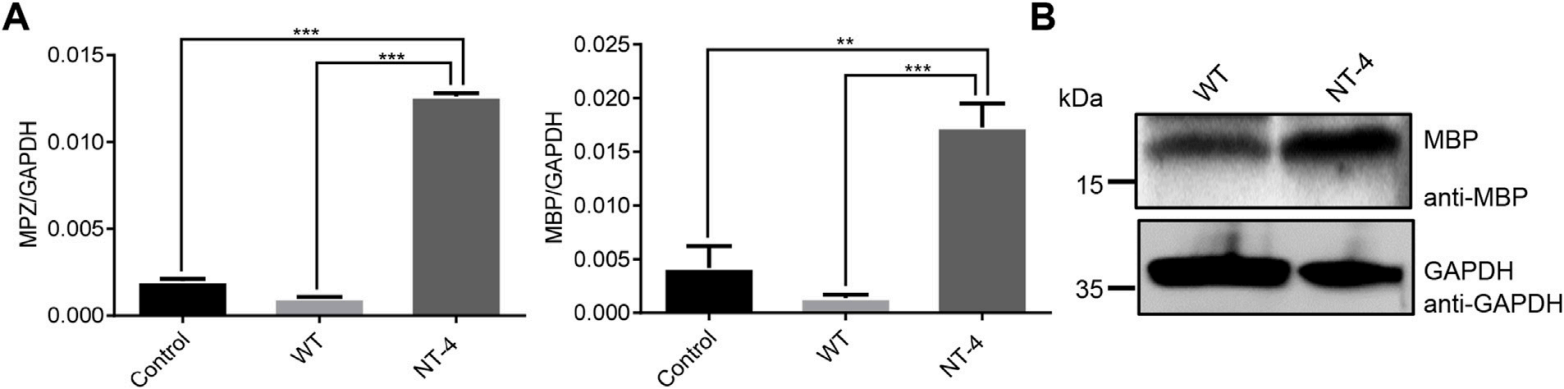
NT-4 contributed to myelination process in HT22 cells. **(A)** The mRNA expression of myelin genes including *MPZ* and *MBP* in HT22 cells were analyzed by qRT-PCR after the treatments with WT and NT-4 extracts. *GADPH* was used as the internal control. For the significant analysis: **p < 0.01 and ***p < 0.001. **(B)** The protein expression of MBP in HT22 cells were analyzed by western blotting after the treatments with WT and NT-4 extracts. GADPH was used as the internal control.

It has been reported that NT-4 is involved in the regulation of myelin synthesis (Guo et al., 2017). To explore the possible role of NT-4 protein in this process, we then examined the effects of NT-4 on the expression of myelin genes, such as *MPZ* (Giese et al., 1992) and *MBP* (Readhead et al., 1990). The results showed a significant increase in the transcription of *MPZ* and *MBP* in NT-4 treatment. Moreover, western blotting further confirmed that MBP protein was highly expressed in NT-4 treatment compared to WT treatment (Figure 5). These results might provide the potential application of the silk material containing NT-4 protein in repair of nerve injury.

### NT-4 induced the potential differentiation of HT22 cells

**FIGURE 6:**
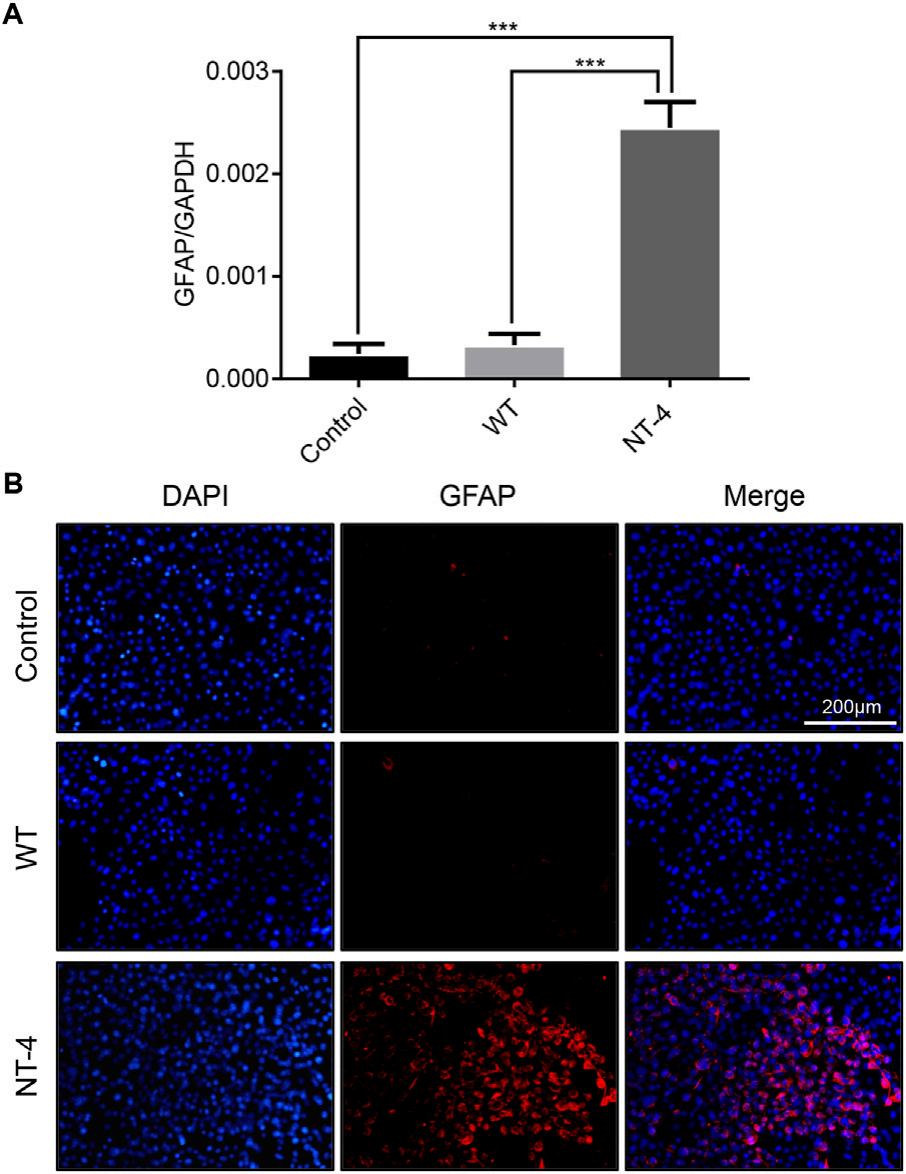
NT-4 induced the potential differentiation of HT22 cells. **(A)** The mRNA expression of astrocyte specific gene *GFAP* in HT22 cells was analyzed by qRT-PCR after the treatments with WT and NT-4 extracts. *GADPH* was used as the internal control. For the significant analysis: ***p < 0.001. **(B)** Immunofluorescence staining of HT22 cells by GFAP antibody after the treatments with WT and NT-4 extracts (red). The nuclei DNA was counterstained with DAPI (blue). Scale bar is 200 μm.

Neurotrophins have the function of regulating the differentiation of neuron cells (Minichiello et al., 1998). We next analyzed the differentiation ability of NT-4 on HT22 cells and detected the expression of *GFAP* (Masood et al., 1993). The qRT-PCR results showed that NT-4 treatment could significantly upregulate the expression of *GFAP* (Figure 6A). Further, the immunofluorescence analysis demonstrated that there was higher red fluorescence signals in NT-4 treatment than that of control and WT treatments (Figure 6B), confirming the induced expression of *GFAP*. These data suggested that NT-4 might promote the potential differentiation of HT22 cells into astrocytes.

### NT-4 promoted peripheral neural cell migration and neurite outgrowth of DRG

**FIGURE 7:**
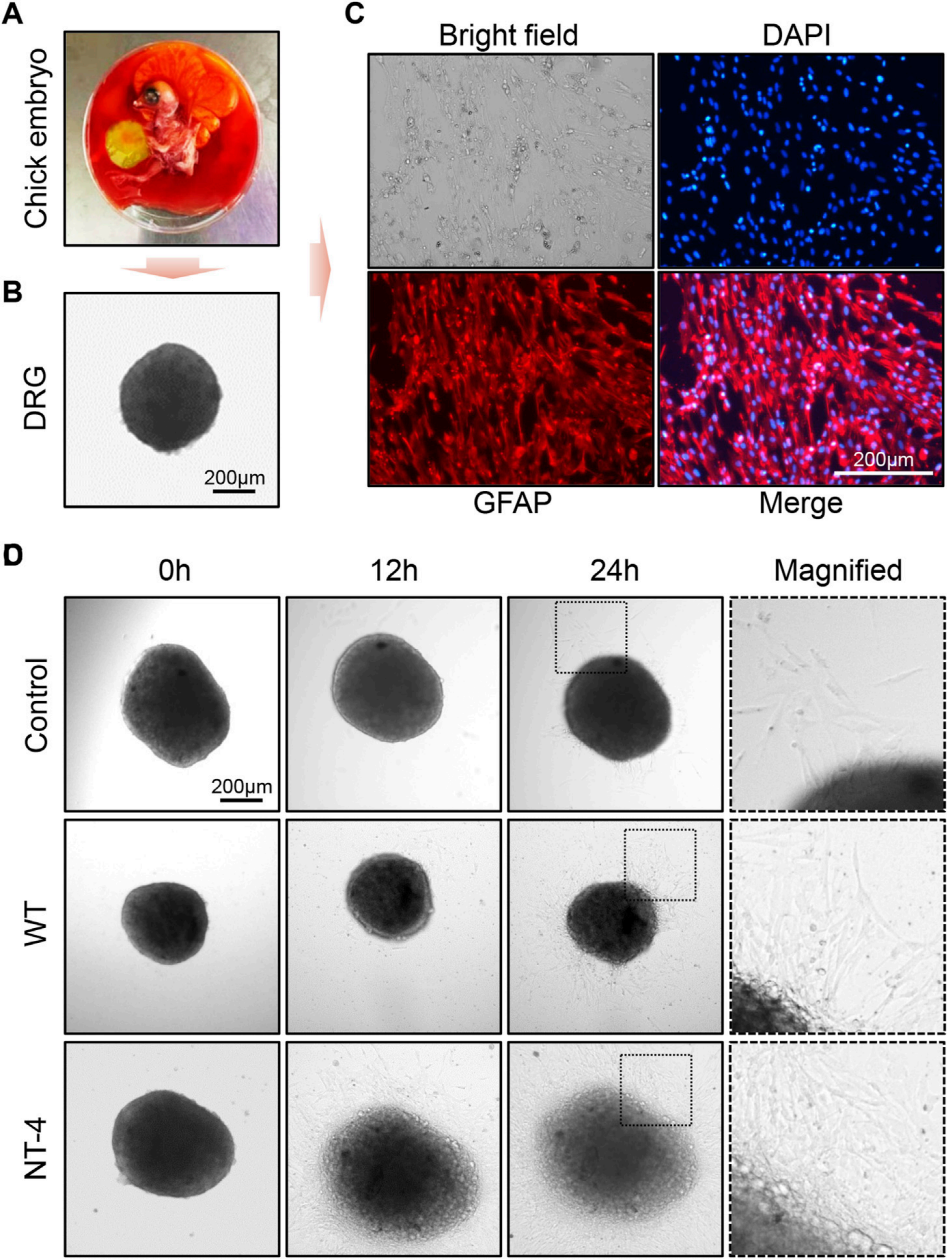
NT-4 promoted peripheral neural cell migration and neurite outgrowth of DRG. **(A)** Chicken embryo was dissected from the egg. **(B)** DRG neurons was isolated from the spinal cord of chicken embryo and observed under a microscope. Scale bar is 200 μm. **(C)** DRGs were cultured in RPMI-1640 complete medium and the peripheral migration cells of DRG were stained by GFAP antibody (red). The nuclei DNA was counterstained with DAPI (blue). Scale bar is 200 μm. **(D)** The peripheral migration progression and neurite outgrowth of DRGs after WT and NT-4 treatments at different time points were analyzed and observed under a microscope. The magnified images showed the distinct speed of DRG outgrowth. Scale bar is 200 μm.

Because DRG is sensitive to the effect of neurotrophins, it has been widely used to test the biological activity of neurotrophins (Tuttle and Matthew, 1995). Previous study also reported that NT-4 can promote neurite outgrowth of embryonic geniculate ganglion (Runge et al., 2012). We herein isolated DRGs from the chicken embryos and placed them in medium for *in vitro* culturing (Figures 7A, B). To identify whether the collected tissue was DRG, we first used the marker protein GFAP to perform an immunofluorescence assay on peripheral migration cells of DRG after incubating for 48 h. It obviously showed that DRG migration cells could be positively stained by GFAP antibody, which confirmed the successful isolation of DRG from chicken embryos (Figure 7C). Upon the treatments with different silkworm cocoon extracts, the results exhibited that compared to the control and WT group, a large number of peripheral neural cells were migrated from DRG with the greatest neurite outgrowth under the condition of the cocoon extract containing NT-4 protein (Figure 7D), demonstrated the high bioactivity of NT-4 protein expressed in silkworm that can efficiently promote peripheral neural cell migration and neurite outgrowth of DRG.

## Discussion

Increasing evidence have shown that the natural silk secreted by silkworm has not only good mechanical properties, but also great biocompatibility and biodegradability, which makes it better to apply in the field of biomedical tissue engineering and injury repair (Lamboni et al., 2015; Das et al., 2021). The natural silk can be fabricated to form various silk biomaterials, such as silk mats, hydrogels, film, sponges, 3D scaffolds, and others (Shi et al., 2021; Wang et al., 2021). With the development of genetic engineering technology in silkworm, the silkworm silk gland has served as a bioreactor model to express various exogenous proteins in order to manufacture different silk protein-based functional biomaterials, which further widens the applications of silk in the medical field (Wang et al., 2018; Wang et al., 2019; Xu et al., 2022).

In this study, we have generated a silkworm strain that can stably express human NT-4 protein in MSG of silkworm, and expectably NT-4 protein can be secreted into the cocoon. The crude extract of NT-4 protein from per Gram of silkworm cocoon could reach to 0.4 mg, which further confirmed the ideal bioreactor of silkworm silk gland to express recombinant foreign proteins (Tomita et al., 2003; Ogawa et al., 2007; Tomita, 2011; Chen et al., 2018). After SDS-PAGE and western blotting analysis, it was interestingly found that the expressed NT-4 protein was able to form mature peptide in silkworm when compared with a commercial standard of active NT-4 protein. However, the active size of NT-4 from silkworm seems to be a litter bigger than the commercial NT-4 standard expressed from *E*. *coli*, this difference might be due to the presence of potential post-translational modification such as glycosylation on NT-4 in eukaryotic silkworm. Future work needs to determine this possibility, and if present, the effect of the modification on NT-4 activity would be another interesting issue worthy to be explored. Anyway, the present data demonstrated that the recombinant NT-4 protein expressed in silkworm would possess similar activity like NT-4 in humans (Ip et al., 1992).

NT-4 protein plays crucial roles in normal nervous system activity, such as maintaining the survival of nerve cells, regulating nerve cell differentiation and directional growth of nerve axons (Conover et al., 1995; Liu et al., 1995; Ernfors, 2001; Runge et al., 2012). In order to determine whether the recombinant NT-4 protein from silkworm was active or not, we first evaluated its activity on nerve cell proliferation. It was clearly shown that the administration of NT-4 protein in neuron HT22 cells was able to promote its proliferation and DNA synthesis. Meanwhile, the presence of NT-4 in HT22 cells could also increase the expression of the neuronal marker genes, such as *NSE*, *TUBB3*, and *MAP2*, which have been implicated in crucial roles in morphogenesis, function, and maintenance of the nervous system (Hisaoka et al., 2003). These data thus supported the biological function of the expressed NT-4 in silkworm during neuron development.

Neuronal myelination process is essential not only for the development of the peripheral nervous system but also for neuronal regeneration after nerve injury (Le et al., 2005). To determine the myelination potential of NT-4 protein, we chose to examine the expression of MPZ and MBP, two important myelin proteins (Guo et al., 2017). MPZ is a major peripheral myelin protein that acts as a homophilic adhesion molecule, while MBP is responsible for adhesion of the cytosolic surfaces of multilayered compact myelin, both of which are essential for compact myelin formation in the peripheral nervous system (Readhead et al., 1990; Giese et al., 1992). After the treatment of HT22 cells by NT-4 protein, the expression of *MPZ* and *MBP* were all induced, which indicated its regulatory signaling for myelin synthesis and potential repair of nerve injury. Therefore, the regulatory mechanisms of NT-4 protein expressed from silkworm for myelin synthesis and nerve repair are worthy to be investigated.

Due to the involvement of NT-4 in neural differentiation (Minichiello et al., 1998), we evaluated this activity of NT-4 by analyzing the expression of astrocyte specific gene *GFAP* (Masood et al., 1993). GFAP is an abundant astrocyte and glial cell cytoskeletal intermediate filament protein, which has been reported as an ideal biomarker for neurological injury (Hayes et al., 2011). The qRT-PCR and immunofluorescence analyses in HT22 cells showed a higher expression of *GFAP* after the application of NT-4 when compared to the WT silk protein. Furthermore, we demonstrated that the NT-4-expressing silk proteins can efficiently promote peripheral neural cell migration of DRG isolated from the chicken embryos. Our data thus provided a possibility that silk materials with the expression of NT-4 could be used as a carrier to deliver the active protein into target cells such as DRG to maintain normal DRG development and regenerate DRG cells after injury.

In summary, this study has developed a novel silk protein-based functional silk material in which the human NT-4 protein was expressed by genetically engineering silk gland of silkworm as a host. Compared to the natural silk protein, the expression of human NT-4 protein had significantly enhanced the bioactivity of silk material, which could promote cell proliferation with no obvious cytotoxicity. Importantly, this functional silk protein was able to induce the differentiation of HT22 cells, promote peripheral neural cell migration and neurite outgrowth of DRG. Based on the excellent mechanical properties and great biocompatibility of silk fibers, our further work is undergoing to fabricate these silk protein-based biomaterials for generating different silk materials such as hydrogels, film, sponges, and 3D scaffolds in order to deliver the active NT-4 protein into target cells and tissues. It is thus expected that these functional silk biomaterials will expand the applications of human NT-4 protein into tissue engineering and neurons regeneration.

## Data Availability

The raw data supporting the conclusion of this article will be made available by the authors, without undue reservation.
