# Supplementary material for: Bioengineered silkworm model for expressing human neurotrophin-4 with potential biomedical application
Source: Front Physiol. 2023 Jan 4;13:1104929. doi: 10.3389/fphys.2022.1104929 (PMC9846172; doi:10.3389/fphys.2022.1104929)
Supplement: Supplementary file 1 [file Table1.DOCX]

**Supplementary Information**

**Table S1. The optimized human NT-4 gene according to the silkworm codon bias.**

ATGCTGCCTCTGCCTTCATGCTCTCTGCCTATCCTGCTGCTGTTCCTGCTGCCTTCAGTGCCTATCGAATCACAACCTCCTCCTTCAACATTGCCTCCTTTCCTGGCTCCTGAATGGGACCTGCTGTCACCTAGAGTGGTGCTGTCAAGAGGTGCTCCTGCTGGTCCTCCTTTGCTGTTCCTGCTGGAAGCTGGTGCTTTCAGAGAATCAGCTGGTGCTCCTGCTAACAGATCAAGAAGAGGTGTGTCAGAAACAGCTCCTGCTTCAAGAAGAGGTGAACTGGCTGTGTGCGACGCTGTTTCAGGTTGGGTGACAGACAGAAGAACAGCTGTGGACCTGAGAGGTAGAGAAGTGGAAGTGCTGGGTGAAGTGCCTGCTGCTGGTGGTTCACCTCTGAGACAATACTTCTTCGAGACAAGATGCAAAGCTGACAACGCTGAAGAAGGTGGTCCTGGTGCTGGTGGTGGTGGTTGTAGAGGTGTGGACAGAAGACACTGGGTGTCAGAATGCAAAGCTAAACAGTCATACGTGAGAGCTCTGACAGCTGACGCTCAAGGTAGAGTGGGTTGGAGATGGATCAGAATCGACACAGCTTGCGTGTGCACACTGTTGTCAAGAACAGGTAGAGCTCACCACCACCACCATCACCATCACTAA
