# Supplementary material for: Bioengineered silkworm model for expressing human neurotrophin-4 with potential biomedical application
Source: Front Physiol. 2023 Jan 4;13:1104929. doi: 10.3389/fphys.2022.1104929 (PMC9846172; doi:10.3389/fphys.2022.1104929)
Supplement: Supplementary file 2 [file Table2.DOCX]

**Supplementary Information**

**Table S2. List of primers used in this study.**

| Gene name | Sequence (5’-3’) |
| --- | --- |
| NT-4-ORF-F  NT-4-ORF-R | GGATCCATGCTGCCTCTGCCTTCATGCTCT |
|  | GCGGCCGCTTAGTGGTGGTGGTGGTGGTGG |
| NT-4-F  NT-4-R | AGAAGGTGGTCCTGGTGCTGG |
|  | TTAGTGGTGGTGGTGGTGG |
| eIF-4a-F  eIF-4a-R | TTCGTACTGGCTCTTCTCGT |
|  | CAAAGTTGATAGCAATTCCCT |
| NSE-F  NSE-R | GGTCCAAGTTCACAGCCAAT |
|  | ATGAACGTGTCCTCGGTTTC |
| TUBB3-F  TUBB3-R | TCCGAGTACCAGCAGTACCA |
|  | TCACTTGGGGCCCTGGGCCT |
| MAP2-F  MAP2-R | ACCAACCACTGCCAGACCT |
|  | GTGGCGGATGTTCTTCAGAG |
| MPZ-F  MPZ-R | ATCGATGAGGTGGGGACCTT |
|  | CCACTATGTCCGGTGGGTTT |
| MBP-F | CATCCTTGACTCCATCGGGC |
| MBP-R | TTGTACATGTTGCACAGCCC |
| GFAP-F | CACGAACGAGTCCCTAGAGC |
| GFAP-R | ATGGTGATGCGGTTTTCTTC |
| GAPDH-F | GGACCTGACCTGCCGTCTAG |
| GAPDH-R | GTAGCCCAGGATGCCCTTGA |
